# Rare Variants in Transcript and Potential Regulatory Regions Explain a Small Percentage of the Missing Heritability of Complex Traits in Cattle

**DOI:** 10.1371/journal.pone.0143945

**Published:** 2015-12-07

**Authors:** Oscar Gonzalez-Recio, Hans D. Daetwyler, Iona M. MacLeod, Jennie E. Pryce, Phil J. Bowman, Ben J. Hayes, Michael E. Goddard

**Affiliations:** 1 Biosciences Research Division, Department of Economic Development, Jobs, Transport and Resources, Bundoora, Victoria, 3083, Australia; 2 Dairy Futures Cooperative Research Centre, Bundoora, Victoria, 3083, Australia; 3 School of Applied Systems Biology, La Trobe University, Bundoora, Victoria, 3086, Australia; 4 Faculty of Veterinary and Agricultural Science, University of Melbourne, Parkville, Victoria, 3010, Australia; Wageningen UR Livestock Research, NETHERLANDS

## Abstract

The proportion of genetic variation in complex traits explained by rare variants is a key question for genomic prediction, and for identifying the basis of “missing heritability”–the proportion of additive genetic variation not captured by common variants on SNP arrays. Sequence variants in transcript and regulatory regions from 429 sequenced animals were used to impute high density SNP genotypes of 3311 Holstein sires to sequence. There were 675,062 common variants (MAF>0.05), 102,549 uncommon variants (0.01<MAF<0.05), and 83,856 rare variants (MAF<0.01). We describe a novel method for estimating the proportion of the rare variants that are sequencing errors using parent-progeny duos. We then used mixed model methodology to estimate the proportion of variance captured by these different classes of variants for fat, milk and protein yields, as well as for fertility. Common sequence variants captured 83%, 77%, 76% and 84% of the total genetic variance for fat, milk, and protein yields and fertility, respectively. This was between 2 and 5% more variance than that captured from 600k SNPs on a high density chip, although the difference was not significant. Rare variants captured 3%, 0%, 1% and 14% of the genetic variance for fat, milk and protein yields, and fertility respectively, whereas pedigree explained the remaining amount of genetic variance (none for fertility). The proportion of variation explained by rare variants is likely to be under-estimated due to reduced accuracies of imputation for this class of variants. Using common sequence variants slightly improved accuracy of genomic predictions for fat and milk yield, compared to high density SNP array genotypes. However, including rare variants from transcript regions did not increase the accuracy of genomic predictions. These results suggest that rare variants recover a small percentage of the missing heritability for complex traits, however very large reference sets will be required to exploit this to improve the accuracy of genomic predictions. Our results do suggest the contribution of rare variants to genetic variation may be greater for fitness traits.

## Introduction

Genome-wide common variants from large scale single nucleotide polymorphism (SNP) genotyping have been used successfully in the last decade to map associated mutations in genome wide association studies (GWAS), and predict future phenotypes for complex traits with genomic prediction. However polymorphisms reaching genome wide significance in GWAS do not explain all of the heritability of complex traits (as reviewed by Manolio et al., [1]). While substantially more genetic variance is captured using all markers simultaneously [2], [3], a proportion of the genetic variance is still not captured by the SNP on widely used arrays. For instance, a joint analyses of 295k common SNPs only explained 45% of the pedigree heritability in human height [2]. For disease traits, the proportion of genetic variance captured can be considerably less—Lee et al., [4] reported that 23% of the variation in liability to schizophrenia was captured by SNPs on a high density array. In livestock, Jensen et al., [5], Haile-Mariam et al., [3] and Roman-Ponce et al., [6] found that 50k common SNPs captured approximately between 80 and 90% of the pedigree heritability for production traits but considerably less for fitness traits such as fertility.

One hypothesis for this “missing heritability” is limited linkage disequilibrium between markers and causative mutations, particularly causative mutations with low minor allele frequency (MAF, rare variants) [1], [7], [8]. The rare variants-common disease theory has been supported by some authors, but not yet rejected or ratified. Some rare variants have already been associated with some complex diseases [9]. However, little or non-significant heritability has been recovered by rare variants in human diseases [10]. Another possibility is that additive genetic variance has been over-estimated in the past, for example in twin studies [11], and hence the ratio of variance explained by the SNPs and the estimates of additive genetic variance from non-genomic information (e.g. twins or pedigree) is always less than one.

The proportion of missing heritability from genotype or sequence data can be estimated in dairy cattle populations, as excellent pedigree recording over long periods of time is available (in our case tracing back to the 1940s), as well as wide spread recording of complex trait phenotypes, such as milk production and fertility. This information enables accurate estimation of genetic variance and heritability. We will use the term ‘missing heritability’ to describe the phenomenon that occurs in these populations when estimates of genetic variance from genomic relationships between individuals calculated from dense SNP chip markers are less than the additive genetic variance estimated from deep pedigree data in these populations. We define the proportion of missing heritability as a population parameter, $\frac{\sigma_{a}^{2}-\sigma_{m}^{2}}{\sigma_{a}^{2}}$, where $\sigma_{a}^{2}$ is the additive variance from pedigree, and $\sigma_{m}^{2}$ is the genetic variance captured by markers.

Substantial whole genome sequence (WGS) data is now available for dairy cattle populations—the 1000 bull genomes project includes key ancestors, that have now been sequenced, from a range of dairy breeds, allowing imputation of variant genotypes, including rare variants, into reference populations genotyped with SNP arrays [12].

WGS data has the advantage that rare alleles can actually be in the data set, so that the proportion of variance explained by the variants is not constrained by LD between markers and the causative mutations. However one of the challenges of using such data is to differentiate between true rare variants and sequencing errors. The primary errors are substitution errors, at rates of 0.5–2.5% per variant [13], [14]. Across the millions of variants detected in cattle (28.3 million reported in Daetwyler et al., [12]), this equates to a very large number of both called variants which are actually mono-morphic sites and genotyping errors. Again, cattle offers a unique opportunity to assess the impact of sequencing error on the proportion of variance explained by rare variants. The 1000 bull genomes project includes a large number of sire-son pairs which can be used to estimate error rates from the proportion of heterozygous sites for rare variants in a sire which result in the rare allele (in the population) being transmitted to the son.

The proportion of variance explained by rare variants in cattle also has important practical implications, as genomic prediction [15] is now routinely used to identify individuals to breed the next generation [16], [17]. Even with extremely large reference populations in dairy cattle (>100,000 animals where SNP effects are estimated), accuracies of genomic predictions using SNP on high density arrays are still considerably below one, with 0.78 the average across a number of traits, and accuracies substantially below this for fitness traits such as fertility [3, 16, 17, 18]. These accuracies are measured as the Pearson correlation between the prediction of the genetic merit of the individuals and the yet-to-be-observed progeny performance. If additional variation could be captured using sequencing variants, the accuracy of genomic prediction could potentially be improved.

The aim of this study was to test whether the hypothesis that sequence variants from coding and potentially regulatory regions, including rare variants can account for the missing heritability previously reported, and to estimate to what extent they can contribute to increasing the predictive ability of genomic selection. First, two strategies to filter ‘true’ rare variants from sequencing errors are proposed. Then, the genetic variance captured by sequence variants in three classes (common, MAF>0.05, uncommon, 0.01<MAF<0.05, and rare, MAF<0.01) was estimated for productive traits and fertility. Finally, the gain in accuracy of genomic prediction from using sequencing variants compared to high-density (HD) SNP genotypes was evaluated using mixed models with genomic relationship matrices.

## Results

### Distinguishing rare variants from sequencing errors

A data set of 3311 Holstein sires, genotyped with either the Bovine SNP50k SNP array or Bovine HD 777k SNP array were imputed to sequence variant genotypes using the Holstein and Jersey animals with WGS data from the 1000 Bull Genomes Project [12], using Beagle3.3 [19]. Only sequence variants in gene coding and flanking regions, in order to reduce computational burden, were imputed. For rare variants, those observed in the 1000 bull genomes data with MAF≤0.01 and appearing in at least two animals were selected (4,444,216 variants in total across the genome). Among those, only 83,856 variants appeared with MAF≤0.01 in the imputed data set (transcript regions), these variants were retained for further analyses. This subset of rare variants is named RV-SET from here onwards.

**Fig 1:**
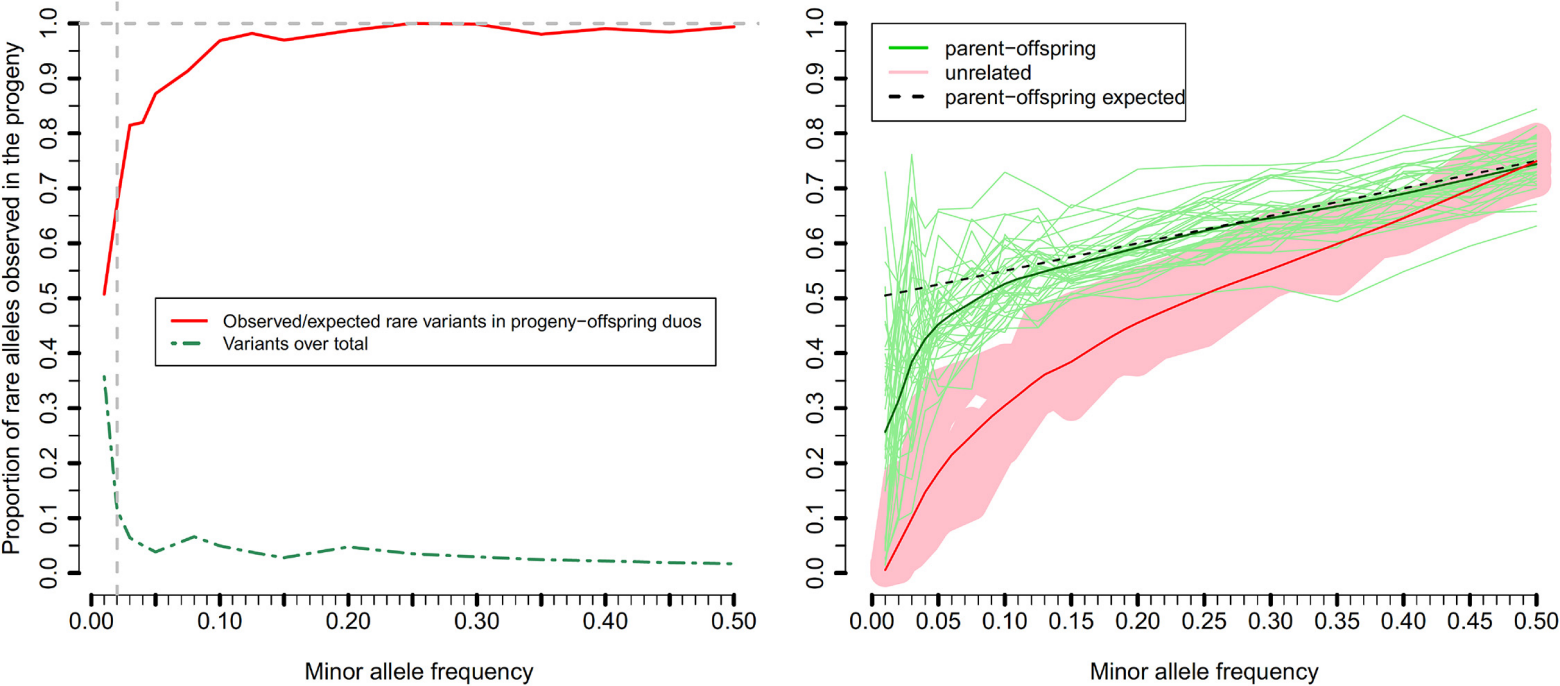
Observed vs expected less common alleles transmitted in 38 sire-son duos(left) and confidence interval of unrelated duos at different MAF (right) in the Bos Taurus autosome 1 (BTA1). Almost 35% of sequence variants had MAF<0.01 (green dashed line in left plot), however 50% of these variants were not observed in the expected proportions in the parent offspring duos (red solid line in left plot). The proportion of transmitted alleles to the progeny was modeled according to the MAF (right plot). Green lines represent each of the duos, and the solid green line is the local weighted regression for the 38 duos. Red shadow represent the confident interval for the same regression when 10 pairs of unrelated animals were evaluated, with the red solid line being the local weighted regression. The dashed black line represents the expected theoretical proportion of transmission for the less frequent allele from sire to son under random mating. At MAF<0.10 we observed that observed proportions of transmission deviated from the theory, which implies that up to 50% of the uncommon and rare variants (at MAF<0.01) are sequencing errors.

With very large numbers of base pairs sequenced, the number of sequencing errors are likely to be considerable. In an attempt to determine what proportion of the rare variants were real and what proportion were sequencing errors, we used Mendelian inheritance patterns in 38 Holstein cattle parent-offspring duos that were in the 1000 bull genomes data set. We investigated what proportion of variants that were heterozygous in the sires were heterozygous in the sons. For rare variants the expectation is close to 0.5, and the expectation increases as MAF increases as there is an increasing probability that the rare allele is also inherited from the dam. When MAF was very low, the proportion of variants where the sire was heterozygous and the son was also heterozygous was much lower than expected, Fig 1. The results imply that for variants that are only observed with less than 4 copies (MAF<0.02) in the data set, up to 50% have a high proportion of genotyping errors or are not true variants. For MAF>0.02, the proportion of double heterozygotes (son and sire) was close to expectation, indicating low rates of genotyping error. As a result of this analysis, we defined a second set of rare variants, those that were validated by the parent-offspring duo method (e.g. those that were heterozygous in a son if they were heterozygous in a sire). This subset of rare variants was called RVvalidated from here onwards.

### Estimates of genetic variance from rare, uncommon and common variants in transcript and regulatory regions

We used lactation average production of fat (FY) and protein (PY), in kg, and milk (MY), in litres, as production traits, and the calving interval (days) as fertility trait. The genetic variance estimated for yield of fat, milk and protein and fertility from the pedigree, BovineHD array and the three classes of sequence variants (common, uncommon and rare), each fitted individually (e.g. separate models), are shown in Table 1. The markers from the Bovine HDarray SNP captured between 81 and 93% of the total genetic variability captured by pedigree. The genetic variances estimated with a total of 675,062 common variants from sequence data (MAF>0.05) were more similar (84–98%) to the estimates obtained with pedigree. On average, common sequence variants captured 3% more genetic variation than HD SNP genotypes when they were the only genetic effect in the model.

**Table 1:** Genetic variance estimates from genomic markers ($\sigma_{g}^{2}$) or pedigree ($\sigma_{a}^{2}$) and their respective standard error (s.e.) for milk, fat and protein yield, and fertility captured from GBLUP models using 3311 Holstein sires. Narrow sense heritability from pedigree is provided, and the proportion of missing heritability from markers was calculated as $(\sigma_{a}^{2}-\sigma_{g}^{2})/\sigma_{a}^{2}$.

Trait	BLUP-PED^1^			GBLUP-HD^2^			GBLUP-Seq^3^		
	***$\sigma_{a}^{2}$***	*se*	*h* ^*2*^	***$\sigma_{g}^{2}$***	*se*	*Missing h* ^*2*^	***$\sigma_{g}^{2}$***	*se*	*Missing h* ^*2*^
**Fat kg**	175	7.2	0.34	143	6.7	18%	146	8.6	17%
**Milk L**	175,355	6,867	0.34	152,554	7449	13%	157,947	10,212	10%
**Prot kg**	127	4.7	0.53	106	5.4	17%	108	7.2	15%
**Fert**	65	4.1	0.20	61	3.8	6%	64	4.5	2%

^1^BLUP model with numerator relationship matrix from pedigree used as a genetic relationship matrix.

^2^GBLUP model with genomic relationship matrix built using 632,003 SNP genotypes;

^3^GBLUP model with genomic relationship matrix constructed using 675,062 SNPs pruned for LD and MAF>0.05.

Next we tested if the variances explained by the different classes of variant in transcript and potential regulatory regions were significantly different. Table 2 shows the increase in log Likelihood from incorporating pedigree, uncommon or rare variants as additional genetic effects into the common variants model, which makes the analyses more meaningful than fitting them separately. For production traits, the inclusion of either the pedigree or rare variants to the common variants model improved the likelihood (*P*<0.005) compared with the model with only common sequence variants. The models including common variants and pedigree had higher likelihood than the models with common and rare variants. Further, incorporating pedigree, common and rare sequence variants together, resulted in the most likely model for FY (*P*<0.05). For fertility, rare variants significantly improved the likelihood compared to the model with only common variants (*P*<0.005), whereas the pedigree did not show any additional improvement. Uncommon variants did not significantly improve the log-likelihood of any model. Using only those rare variants validated from sire-son transmission did not increase the likelihood either (results not shown).

**Table 2:** Level of statistical significance of the log-likelihood tests from GBLUP models incorporating different sources of genetic information against GBLUP model incorporating only common variants^1^.

	Common variants and pedigree^2^	Common variants and rare variants^3^	Common variants, pedigree and uncommon variants^4^	Common variants, pedigree and rare variants^5^
**Fat kg**	**	**	N.S.	† ^(b)^
**Milk L**	**	*	N.S.	N.S.^(b)^
**Prot kg**	**	**	N.S.	N.S.^(b)^
**Fert**	N.S.	**	N.S.	** ^(b)^

^1^GBLUP-Seq model: genomic relationship matrix constructed using 675,062 SNPs pruned for LD<0.999 and MAF>0.05;

^2^as (^1^) plus the polygenic effect

^3^as (^1^) with an additional random effect with genomic relationship matrix constructed from 83,856 variants with MAF<0.01 detected in 429 sequenced animals;

^4^as (^2^) with an additional random effect with genomic relationship matrix constructed from variants with 0.01<MAF<0.05 extracted from the imputed-sequence data set;

^5^as (^2^) with an additional random effect with genomic relationship matrix constructed from 83,856 variants with MAF<0.01 detected in 429 sequenced animals;

**P<0.005

*P<0.025

^†^P<0.05

^(b)^Statistical test against model ^2^.

Common sequence variants captured most of the genetic variance for all traits when pedigree, common, uncommon and rare variants were fitted jointly in the model for production traits (Table 3), accounting for 76–84% of the total genetic variance. Pedigree accounted for between 14 and 23% of the total genetic variance, whereas rare variants accounted for between 0 and 3% of total genetic variance. Uncommon variants did not contribute to the genetic variance in the joint analyses. For fertility, rare variants explained a larger proportion of the total genetic variance compared to production traits (14%), and the pedigree did not provide any additional information.

**Table 3:** Posterior mean estimates (standard errors within brackets) for proportion of genetic variance for milk, fat and protein yield, and fertility captured from the GBLUP model fitting jointly: pedigree and common and rare variants, using 3311 Holstein sires.

	Pedigree	Common variants	Uncommon variants	Rare variants	Total additive variance
**Fat kg**	14% (2)	83% (10)	0% (0)	3% (1)	147
**Milk L**	23% (0.1)	77% (0.1)	0% (0)	0% (0)	157919
**Prot kg**	23% (5)	76% (14)	0% (0)	1% (1)	108
**Fert**	2% (0.4)	84% (16)	0% (0)	14% (5)	63.9

These results imply that, although most of the genetic variance can be explained by common sequence variants in these regions, the pedigree still explains some additional genetic variability of the traits studied here, and rare variants explain more variation for fitness traits such as fertility.

### Accuracy of genomic prediction

We evaluated the accuracy of genomic prediction that could be achieved with GBLUP [20], [21], with the three classes of variants and the Bovine HD array, with the BLUP model with only pedigree information as benchmark. The surrogate for the accuracy of genomic prediction was the correlation of the genomic predictions and phenotypes in a validation set of 465 bulls (these were the youngest bulls in the data set, and their phenotypes were never used in the derivation of genomic predictions).

All models incorporating genomic information were more accurate than the model using only pedigree. Accuracy of genomic prediction using the BovineHD SNP genotypes or sequence variants (regardless of class of variant) were very similar, Table 4. There were slight (0.01–0.02) improvements for fat yield and milk yield using sequence variants in comparison to BovineHD SNP genotypes.

**Table 4:** Pearson correlation (cor), slope coefficient for the linear regression and mean square error^7^ (MSE) between observed and predicted daughter yield deviation for fat, milk and protein yield, and fertility from different GBLUP models. Training set consisted of 2832 animals and there were 465 animals in the validation set.

Trait	BLUP^1^			GBLUP-HD^2^			GBLUP-Seq^3^			GBLUP-Seq-Uncommon^4^			GBLUP-Seq-RV-SET^5^			GBLUP-Seq-RVvalidated^6^		
	*cor*	*slope*	*MSE*	*cor*	*slope*	*MSE*	*cor*	*slope*	*MSE*	*cor*	*slope*	*MSE*	*cor*	*Slope*	*MSE*	*cor*	*slope*	*MSE*
**Fat kg**	0.46	0.79	0.98	0.56	0.96	0.83	0.57	0.94	0.82	0.57	0.94	0.82	0.57	0.95	0.82	0.57	0.95	0.82
**Milk l**	0.52	0.86	0.81	0.61	0.92	0.65	0.63	0.93	0.63	0.63	0.93	0.63	0.63	0.93	0.63	0.63	0.93	0.63
**Prot kg**	0.57	0.89	0.77	0.65	0.98	0.64	0.65	0.97	0.64	0.65	0.97	0.64	0.65	0.97	0.64	0.65	0.97	0.64
**Fert**	0.36	0.87	3.02	0.43	1.13	2.80	0.42	1.12	2.80	0.42	1.12	2.80	0.43	1.17	2.80	0.43	1.17	2.80

^1^ BLUP model with pedigree numerator relationship matrix;

^2^ G-BLUP model with genomic relationship matrix built using 632,003 SNP genotypes;

^3^ G-BLUP model with genomic relationship matrix constructed using 675,062 SNPs pruned for LD and MAF>0.05;

^4^ G-BLUP model with genomic relationship matrix for common variants constructed using 675,062 SNPs pruned for LD and MAF>0.05 and a genomic relationship matrix constructed from variants with 0.01<MAF<0.05 extracted from the imputed-to-sequence data set;

^5^ G-BLUP model with genomic relationship matrix for common variants constructed using 675,062 SNPs pruned for LD and MAF>0.05 and genomic relationship matrix for rare variants constructed from 83,856 variants with MAF<0.01 detected in 429 sequenced animals;

^6^ G-BLUP model with genomic relationship matrix for common variants constructed using 985,757 SNPs pruned for LD and MAF>0.05 and genomic relationship matrix for rare variants constructed from 20,648 confirmed rare variants detected in 38 sire-son duos;

^7^ MSE is expressed as units of additive genetic standard deviations. All models included a polygenic effect.

Rare variants did not increase the predictive ability in comparison to common sequence data regardless of whether they were validated through duos or not.

## Discussion

In our dairy cattle population, relationships defined by SNPs and also by sequence variants in annotated regions captured less genetic variance than pedigree. One potential issue here is that the genetic variances estimated by markers and pedigree may not be strictly equivalent because using pedigree estimates the genetic variance among founders, whereas the genetic variance estimated from the SNP genotypes or sequences are estimates of the genetic variance in the modern (genotyped or sequenced) population. It should be noted nonetheless that we do in fact have many of the founders of the population either genotyped with BovineHD and in the sequence data set [12]. Further, the SNPs on the Bovine HD array are ascertained to have high MAF, and therefore are unlikely to be in LD with causal mutations with low MAF [22]. The limits of LD between common and rare variants were studied by Wray [23]. The LD between rare variants and common SNPs tends to zero as the MAF of the rare variant approaches to zero. These findings are supported by an example on human chromosome 21 [24], and also in S1 Fig of our data. The proportion of missing heritability we observe from SNP array genotypes is in agreement with previous studies [3]. [4]. [5].

We overcome this limitation by fitting the pedigree and sequence variants from transcript and regulatory regions simultaneously, and tested the hypothesis that the additional variance component is significant by investigating the change in log likelihoods. We found that WGS explained only part of this missing heritability (Table 2). The recovery of the missing heritability was minor for PY and MY, showing that common WGS variants may track little additional information that is not tracked from HD SNP genotypes for these traits. We did observe an increase on the heritability (3% for FY and up to 14% for fertility) captured by variants with very low frequencies. Fertility is a fitness trait and natural selection may have driven deleterious QTLs to extreme frequencies where they cannot be in high LD with individual SNPs on the SNP chip.

These figures (3% and 14%) are expected to be under-estimates because we only included sequence variants in gene coding and flanking regions within 2 kb of annotated regions. Causal variants for complex traits probably occur throughout the genome [25, 26, 27] but they are more likely close to genes, which explain most of the genetic variance [10]. Sequence variants in unannotated regulatory regions might explain additional variance. However, the proportion of variance explained by these mutations, or at least those not in LD with common variants, must be relatively small in our population. The reason is that similar genetic variance was captured when using only variants in annotated regions compared to pedigree and HD SNP genotypes (Table 1).

Here, we demonstrated that rare variants are capturing variation different from that captured by common variants, and possibly include variation from rare deleterious effect. It is desirable to recognize rare mutations to drive them to higher frequency in populations under artificial selection if they are favorable or remove them if they are deleterious.

This is the first time, to the best of our knowledge, that mixed models accounting simultaneously for pedigree, common and rare sequence variants have been used to estimate the heritability explained by each. Many studies in humans have reported that the genetic variance explained by SNPs is only 1/3 to ½ that estimated from pedigree using analyses that fitted only one variance component at a time. Yang et al [2] showed the missing heritability could be due to low MAF in causal variants but offered no direct proof of this. Other studies have offered some insight, for instance, Liu and Leal [28] estimated that rare variants in the ANGPTL4 gene contribute at least 1.63% of the overall heritability of triglyceride in blood. An et al., [29] estimated a larger heritability in circulating adiponectin explained by rare variants, between 6 and 18% of the variance in Hispanic and African American populations.

We would not expect the gap between genetic variance estimated from pedigree and WGS to be as great in Holstein cattle as in humans. Holsteins have a smaller recent effective population size (around 100) than human populations and hence fewer rare variants are expected in the Holstein population due to inbreeding, which flattens the allele frequency spectrum. The lower recent effective population size also leads to longer range LD and hence, in cattle, a rare causal variant may be predicted from a linear combination of SNPs spread over a large genomic region.

**Fig 2:**
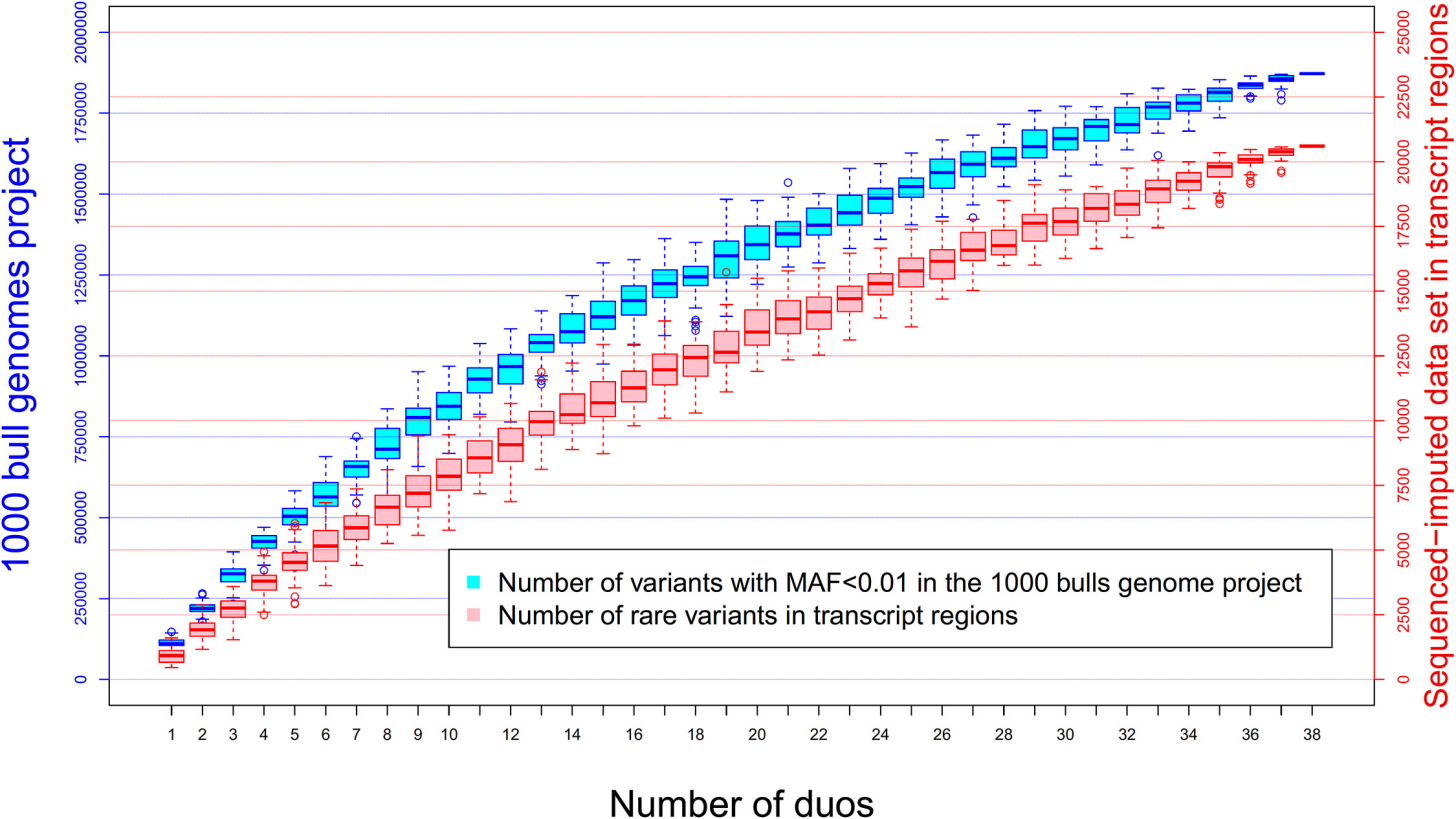
Number of variants detected by number of parent-offspring duos. Boxplot for the occurrence of variants with MAF<0.01 detected from 38 sire-son duos (blue). The boxplots in red show how many of them were present in transcript regions. Each boxplot is constructed from 50 replicates of random samples of a given number of duos (from 1 to 38). Solid lines are the corresponding quadratic regression for the number of rare variants discovered in the Australian Holstein population according the number of duos used. The regression equation for the total number of rare variants (y) according to the number of duos (x) was y = 108432+80557x-926x^2^. The regression equation equivalent for the number of rare variants in transcript regions was y = 653+775x-6.7x^2^. This means that we would need 44 duos for detecting most of the rare variants along the genome, which number is projected to be 1,860,826. Among these 23,000 are expected to be present in transcript regions, and 58 parent offspring duos would be necessary to detect them.

The larger genetic variance captured by WGS data did not translate into higher accuracy of genomic prediction. That is, including the rare variants did not improve the predictive ability of the models. There are at least 4 possible reasons for this: 1) The increase in variance explained is small; 2) the small size of the training population limits the accuracy with which the effect of rare variants can be estimated; 3) long LD in Holsteins means that common SNPs can trace signals from a rare QTL, and 4) rare variant genotypes are poorly imputed. For 3), this is particularly the case here as our validation population was only one generation removed from the training or reference population. We would expect greater benefit from the sequence data if the prediction was tested in a validation population less closely related to the reference population [30], [31]. That is, if the causal mutations are in the data, we would expect more robust prediction of breeding value because we would not be relying on long distance LD between SNPs and causal variants which may decay rapidly over generations [32], [33]. To capture the benefit of this increased robustness, it may be necessary to use statistical methods that give increased emphasis to variants close to the causal variants [34], rather than estimate small effects for all variants as in G-BLUP [25], [35], [36], [37], or GWAS which may cause synthetic association with common variants [38], [39]. The accuracy of estimating individual sequence variant effects is limited by the accuracy with which rare variants are imputed from HD SNP data. Unfortunately, the accuracy of imputation for rare alleles is low at present [40]. S2 Fig shows a large range in imputation accuracy across MAF for rare variants. The lower the MAF the lower the accuracy. Nonetheless, many rare variants are imputed at accuracies close to 1. One reason why some rare alleles are hard to impute accurately might be that many of them are sequencing errors, or the rate of genotyping error is high. We used the transmission of rare alleles from sire to son to show that as the MAF falls below 0.1 the fraction of rare alleles rises towards 50% (Fig 2). It is possible to use this transmission to validate rare alleles by deleting from the data all alleles not so validated. The number of rare variants that we were able to distinguish from sequencing error with our duo approach increased with the number of duos utilized (Fig 2). This curve was fitted with a non-linear regression detailed in S1 Text. The resulting non-linear function of the number of confirmed rare variants (*y*) was *y* = *T*(1-*e* ^*-kN*^), where *T* was the total number of rare variants, *N* was the number of duos available and *k* depends on the allele frequencies of rare variants. The value of *T* was estimated at 2.19 million of total rare variants in the Holstein genome, and 29,930 rare variants in transcript and up or down-stream regions like those used here. However, we didn’t observe larger accuracies when using only these validated rare variants. It would be necessary to have around 150 duos to confirm most of the rare variants present in these regions in the Holstein population under study. In this study, only about 70% of total rare variants were validated. However, even a 50% increase in the number of true rare variants would only be expected to increase the modest variance ascribed to them by 50%. Further research is needed to distinguish rare variants from sequencing errors using an alternative method to the parent-offspring duos strategy, because rare variants validated in this way track familiar relationships as well as possible rare causal variants.

## Materials and Methods

### Sequence data preparation

As part of the 1000 Bull Genomes Project Run 3, a total of 429 individuals of 15 breeds were re-sequenced with Illumina and SOLiD technology. In many breeds the sequenced ancestors were key ancestors that captured the most genetic variation, chosen by algorithms similar to Druet et al., [41].

Raw re-sequence reads were filtered based on chastity score and trimmed based on quality scores. Fastq files were then aligned to the reference bovine genome assembly UMD3.1 using BWA [42]. All BAM files were analysed simultaneously using Samtools-0.1.18 mpileup to call variants [43]. Variants were filtered to reduce the incidence of false positives. Variants were discarded if they had: two or more alternative alleles, no observations of the alternative allele on either the forward or reverse reads, an overall quality score (QUAL) of <20, a mapping quality (MQ) score of <30, a read depth of <10 or more than median plus 3 SD read depth, >10% opposing homozygotes between parent and offspring pairs, or the same bp position (*e*.*g*. SNP overlapping with indel). In addition, we also filtered variants for proximity: when an indel was within 10 bp of another indel, the indel with a lower QUAL score was removed; when any variant was within 3 bp of another variant, the lower QUAL variant was removed. The filters were implemented by extending the python VCF file parser PyVCF (https://github.com/jamescasbon/PyVCF/).

The Genome Analyzer Tool Kit [44] was used to convert Phred score genotype probabilities in the filtered VCF file to true probabilities. In turn, these probabilities were utilized by BEAGLE [19] to impute missing genotypes and correct low probability genotype calls arising from incomplete coverage. Concordance of re-sequence genotypes with Bovine HD chip genotypes was calculated as the proportion of identical genotypes pre and post the BEAGLE step. Opposing homozygotes were calculated as the proportion of non-matching homozygotes between parent-offspring pairs and collected per pair and per locus. SNPs and indels were annotated with predicted functional consequences using NGS-SNP [12], [45]. The sequenced variants that were identified during annotation were restricted to the coding regions and potentially regulatory regions (including 3’ and 5’ untranslated gene regions and ±2000 bp up- and down-stream of genes) (*i*.*e*. totalling 2,785,440).

### Imputation to sequence

A data set of 3311 Holstein bulls were genotyped either with the Illumina Bovine 54K SNP array or the Illumina Bovine HD SNP array (777K SNP). After quality control of the genotype data following Erbe et al., [35], 43,425 and 632,002 segregating SNP remained. All animals with the 54K genotypes were imputed to the HD SNP using Beagle 3 [19]. Only the 122 Holstein and 26 Jersey animals with WGS data from the Run 3 of the 1000 bull genomes project were used to impute the subset of 2,785,440 sequence SNPs and indels in transcript and regulatory regions into the 3311 Holstein bulls.

### Pruning of uncommon and common variants in the sequence data

Variants were classified as uncommon if 0.01< MAF < 0.05 and common if they had MAF≥0.05. Also one of each pair of variants that were found to be in complete LD (r^2^ genotypic correlation >0.999) was removed. This pruning was carried out with PLINK software [46]. The number of common and uncommon variants retained were 675,062 and 102,549, respectively.

### Detection of rare variants

Two procedures were implemented to select rare variants. The first one selected those rare variants that appeared at least twice in the WGS from the 433 bull genomes data set and had MAF<0.01. Then, those that were still present with MAF<0.01 in the imputed data were allocated to a rare variant set (RV-SET) with 83,856 variants in total. This procedure does not confirm the rare variants or distinguish them from sequencing errors. A second procedure validated these rare variants utilizing duos of sires and their sons, and kept only those that were present in both individuals of the duo (RVvalidated). If both of them carry the same rare mutation, it is likely that it is a ‘true’ rare variant rather than a sequencing error. A sire that is homozygous for the less frequent allele will transmit it to 100% of the progeny. However, if the allele is very rare, homozygous individuals for this variant will be observed very infrequently, and therefore many rare variants would be missing. Rare variants appear more frequently in heterozygous form. Here, the less common allele with some MAF, is transmitted to the progeny in a proportion of 50%. Therefore, the theoretical probability of observing at least one rare variant in the son in the loci along the genome is:

$$p(a_{progeny}|Aa_{sire})=0.5(1-MAF)+MAF$$

We used 38 sequenced Holstein sire-son duos to calculate the proportion of loci at which the sire was heterozygous and his son presented at least one rare allele for different MAF along all chromosomes. The same procedure was undertaken at other 10 pairs of unrelated Holstein individuals selected at random, as a control sample. These proportions were modeled via a local weighted regression at different MAF. Local weighted regression is a nonparametric approach to fitting curves to data based on smoothing [47]. This method approximates the relationship between the proportion of loci sharing the less common allele in both sire and son (response variable) and the MAF (explanatory variables) locally by a smooth curve based on a non-parametric function, using locally weighted least squares. Weights are assigned such that points close (in the Euclidean distance) to the predictor value of interest receive a higher weight. For simplicity, fitting was such that one fifth of the points in the plot were allowed to influence the smoothing at each value. The regressions were computed using the *loess* function built in R software [48], and were compared to the theoretical expected proportion under the null hypothesis of no mutation and no sequencing errors in the control set of 10 unrelated pairs.

Then, the variants with MAF<0.01 that appeared in both the sire and the son in any of the duos were proposed as ‘true’ rare variants. These ‘true’ rare variants were extracted from the imputed-to-sequence data set. Those which were still observed with MAF<0.01 were kept for further analyses, and recognized as validated rare variants (20,648).

### Phenotypes

Four different complex traits were analyzed: lactation average of kg of fat (FY), litres of milk (MY) and kg of protein (PY) yields, as production traits, and calving interval, in days, as a fertility trait. Daughter trait deviations were used as phenotypes, which are the daughter average performance previously adjusted by environmental effects. Phenotypic correlation between traits, as well as histograms are shown in S3 Fig. Heritabilities and genetic correlations between traits are shown in S2 Text.

### Estimation of genetic variance

The G-BLUP model [2], [49] was implemented to obtain the genetic variance estimates. The underlying statistical model was

y=1μ+Zg+e

In this model, the *i* ^th^ component of the **y** vector is the phenotypic value of the *i* ^th^ animal used for prediction. Then, **1** was an *n*-row vector of ones (*n* being the number of records), and *μ* is the overall mean; **g** was the vector of additive genetic merits of the animals, assumed to be multivariate normal as $g\sim N(0,\sigma_{g}^{2}G)$, with **G** the genomic relationship matrix of all *n* animals calculated as in Yang et al., [2]. Here, $\sigma_{g}^{2}$ was the additive genetic variance among sires. The matrix **Z** is an ^(n×n)^-incidence matrix, whose rows consist of unit vectors with one component being 1 and all the others zero, indicating the respective positions of animals in the **g**-vector of genetic values. Finally, $e\sim N(0,\sigma_{e}^{2}D)$ is the residual term, where $\sigma_{e}^{2}$ is the residual variance and **D** a diagonal matrix with weights on the residual variance according to the number of effective numbers of the sire at calculating his phenotype [50]. The covariance matrix **G** was calculated as in Yang et al., [2].

Three different models per trait were used in which the only difference was the construction of the **G** matrix:

1 GBLUP-HD. constructed with 580,125 common SNPs (MAF>0.05) from the BovineHD Illumina Beadchip,
2 GBLUP-Seq. constructed with the 675,062 common variants from pruning the sequence data for LD>0.999 and MAF<0.05.
3 BLUP-PED. the **G** matrix was assumed to be the numerator relationship matrix made up from pedigree relationships. The pedigree contained 8978 animals tracked up to 7 equivalent generation in the Holstein data set. This is usually named the **A** matrix and the model is equivalent to the commonly known as a BLUP model in animal breeding.

Variance components were estimated via restricted maximum likelihood using ASReml 3 [51].

#### Log likelihood ratio test

Since the common-variants complex-traits theory is well-established, we tested if the inclusion of pedigree and rare variants further improved the statistical fitting through log likelihood ratio test as

$$\begin{array}{l} \chi_{df=1}^{2}=-2\text{log}\frac{\text{likelihood for common variants model}}{\text{likelihood for alternative model}}\\ =-2\text{log}Lik(\text{common variants})+2\text{log}Lik(\text{alternative model}) \end{array}$$

We assumed a significance threshold of P<0.005 for the $\chi_{df=1}^{2}$ distribution, which corresponds to a value of 6.635.

### Genome-wide prediction using sequence and rare variants data

The predictive ability of different sorts of genomic information was assessed using cross validation. The data set was split into reference and validation sets. The former included 2832 Holstein sires, whereas the latter was created with the 465 youngest animals in the data set.

The underlying statistical model to predict data in the validation set included a genomic and a polygenic effect [3] as,

$$y=w\mu +Z_{g}g+Zu+e$$

In this model, **g** was the genomic component assumed to be distributed as $g\sim N(0,\sigma_{g}^{2}G)$. We compared two different models differing in the marker subsets used to calculate **G** (described in models 1–2 above). Then, $u\sim N(0,\sigma_{a}^{2}A)$ was the polygenic effect with **Z** being a corresponding incidence matrix. All other components were as described previously. Model 3 above with corresponding reference and validation sets was considered as the benchmark model.

Additionally, three joint analyses of common and uncommon or rare variants from sequence data were implemented using the following model:

4 **y = w** *μ*+**Z**_*seq*_ **g**_*seq*_+**Z**_*uncommon*_ **g**_*uncommon*_+**Zu**+**e**
5 **y = w** *μ*+**Z**_*seq*_ **g**_*seq*_+**Z**_*rv−set*_ **g**_*rv−set*_+**Zu**+**e**
6 **y = w** *μ*+**Z**_*seq*_ **g**_*seq*_+**Z**_*rv−validated*_ **g**_*rv−validated*_+**Zu**+**e**

where **g**_*seq*_, **g**_*uncommon*_, **g**_*rv-set*_ and **g**_*rv-validated*_ were the vector of additive genetic merits for the common, uncommon, rare variants from sequence data, and rare variants from duos, respectively, assumed to be multivariate normal as $g_{\cdot }\sim N(0,\sigma_{g_{\cdot }}^{2}G_{\cdot })$, with **G**. being the corresponding genomic relationship matrix for each set of variants constructed as described above, and $\sigma_{g_{\cdot }}^{2}$ the genetic variance explained by such set of markers. All other components were as described previously.

Predictive ability was assessed using the metrics of Pearson correlation, slope of the linear regression and predicted mean squared error between predicted and observed daughter yield deviations in the validation set.

## Supporting Information

S1 Fig Heat map between rare variants and common SNPs (1/5 of SNPs uniformly distributed) in the Bos Taurus autosome 1 (BTA1). (TIF)

S2 Fig Box plots for imputation accuracy estimated from Beagle [19] for variants with (MAF<0.01) according to their minor allele frequency in the imputed data set of 3311 sires. Density distribution for imputation accuracy is shown in the plot for each group of MAF.
(TIFF)

S3 Fig Summary plot of phenotypes. The histograms and corresponding density functions are plotted in the diagonal. On the upper diagonal the value of the phenotypic correlation plus the result of a correlation test ^(^***P<0.001). On the lower diagonal, the bivariate scatterplots, with a fitted line.
(TIFF)

S1 Text Probability to validate rare variants with N sire-son duos. (DOCX)

S2 Text Pedigree heritability (diagonal), phenotypic covariances (up-diagonal) and pedigree genetic correlations (low-diagonal) for traits in the analyses. (DOCX)

## References

[1] Manolio TA, Collins FS, Cox NJ, Goldstein DB, Hindorff LA, Hunter DJ, et al (2009) Finding the missing heritability. Nature 461: 747–753. 10.1038/nature08494 19812666 PMC2831613

[2] Yang JB. Benyamin BP, McEvoy S, Gordon AK, Henders AK, Nyholt DR, et al (2010) Common SNPs explain a large proportion of the heritability for human height. Nat Genet 42: 565–569. 10.1038/ng.608 20562875 PMC3232052

[3] Haile-Mariam M, Nieuwhorf GJ, Beard KT, Konstantinov KV, Hayes BJ (2013) Comparison of heritabilities of dairy traits in Australia Holstein Friesian cattle from genomic and pedigree data and implications for genomic evaluations. J Anim Breed Genet 130(1): 20–31. 10.1111/j.1439-0388.2013.01001.x 23317062

[4] Lee SH, DeCandia TR, Ripke S, Yang J. The Schizophrenia Psychiatric Genome-Wide Association Study Consortium (PGC-SCZ), The International Schizophrenia Consortium (ISC), et al (2012) Estimating the proportion of variation in susceptibility to schizophrenia captured by common SNPs. Nat. Genet. 44: 247–250. 10.1038/ng.1108 22344220 PMC3327879

[5] Jensen J, Guosheng S, Madsen P (2012) Partitioning additive genetic variance into genomic and remaining polygenic components for complex traits in dairy cattle. Genet Sel Evol 13: 44. 10.1186/1471-2156-13-44 PMC3472176 22694746

[6] Román-Ponce SI, Samoré AB, Dolezal MA, Bagnato A, Meuwissen THE (2014) Estimates of genetic heritability for complex traits in Brown Swiss cattle. Genet Sel Evol 46: 36 10.1186/1297-9686-46-36 24898214 PMC4118788

[7] Gibson G (2012) Rare and common variants: twenty arguments. Nat Rev Genet 13: 135–145. 10.1038/nrg3118 22251874 PMC4408201

[8] Zuk O, Schaffner SF, Samocha K, Do R, Hechter E, Kathiresan S, et al (2014) Searching for missing heritability: Designing rare variant association studies. PNAS 17: E455–E464. 10.1073/pnas.1322563111 PMC3910587 24443550

[9] Cirulli ET, Goldstein DB (2010) Uncovering the roles of rare variants in common disease through whole-genome sequencing. Nat Rev Genet 11: 415 10.1038/nrg2779 20479773

[10] Gusev A, Hong Lee S, Trynka G, Finucane H, Vilhjálmsson BJ, Xu H, et al (2014) Partitioning heritability of regulatory and cell-type-specific variants across 11 common diseases. Amer. J. Human Genet 95: 535–552. 25439723 10.1016/j.ajhg.2014.10.004 PMC4225595

[11] Zuk O, Hechter E, Sunyaev SR, Lander ES (2012) The mystery of missing heritability: genetic interaction create phantom heritability. PNAS 109(4): 1193–1198. 10.1073/pnas.1119675109 22223662 PMC3268279

[12] Daetwyler HD, Capitan A, Pausch H, Stothard P, van Binsbergen R, Brondum RF, et al 2014 Whole genome sequencing of 234 bulls facilitates mapping of monogenic and complex traits in cattle. Nat Genet 10.1038/ng.3034 25017103

[13] Meacham F, Boffelli D, Dhahbi J, Martin DI, Singer M, Pachter L (2011) Identification and correction of systematic error in high-throughput sequence data. BMC Bioinformatics 12: 451 10.1186/1471-2105-12-451 22099972 PMC3295828

[14] Loman NJ, Misra RV, Dallman TJ, Constantinidou C, Gharbia SE, Wain J, et al (2012) Performance comparison of benchtop high-throughput sequencing platforms. Nat. Biotechnol 30 (5): 434–439. ISSN 1087-0156. 10.1038/nbt.2198 22522955

[15] Meuwissen THE, Hayes BJ, Goddard ME (2001) Prediction of total genetic value using genome-wide dense marker maps. Genet 157: 1819–1829. 10.1093/genetics/157.4.1819 PMC1461589 11290733

[16] Lund MS, de Roos APW, de Vries AG, Druet T, Ducrocq V, Fritz S, et al (2011) A common reference population from four European Holstein populations increases reliability of genomic predictions. Genet Sel Evol, 43: 43 10.1186/1297-9686-43-43 22152008 PMC3292506

[17] Wiggans GR, VanRaden PM, Cooper TA (2011) The genomic evaluation system in the United States: past, present, future. J Dairy Sci 94: 3202–3211. 10.3168/jds.2010-3866 21605789

[18] VanRaden PM, Null DJ, Sargolzaei M, Wiggans GR, Tooker ME, Cole JB, et al (2013) Genomic imputation and evaluation using high-density Holstein genotypes. J Dairy Sci 96(1): 668–678. 10.3168/jds.2012-5702 23063157

[19] Browning BL, Browning SR (2009) A unified approach to genotype imputation and haplotype-phase inference for large data sets of trios and unrelated individuals. Am J Hum Genet 84: 210–223. 10.1016/j.ajhg.2009.01.005 19200528 PMC2668004

[20] Hayes BJ, Bowman PJ, Chamberlain AJ, Goddard ME (2009) Invited review: Genomic selection in dairy cattle: progress and challenges. J Dairy Sci 92: 433–443. 10.3168/jds.2008-1646 19164653

[21] VanRaden PM, Van Tassell CP, Wiggans GR, Sonstegard TS, Schnabel RD, Taylor JF, et al (2009) Invited review: Reliability of genomic predictions for North American Holstein bulls. J. Dairy Sci. 92: 16–24. 10.3168/jds.2008-1514 19109259

[22] de los Campos G, Sorensen D, Gianola D (2015) Genomic Heritability: What Is It? PLoS Genet 11(5): e1005048 10.1371/journal.pgen.1005048 25942577 PMC4420472

[23] Wray NR (2005) Allele frequencies and the r^2^ measure of linkage disequilibrium: impact on design and interpretation of association studies. Twin Res Hum Genet 8(2): 87–94. 15901470 10.1375/1832427053738827

[24] Lopez de Maturana E, Ibañez-Escriche N, González-Recio O, Marenne G, Mehrban H, Chanock S, et al (2014) Next generation modelling in GWAS: comparing different genetic architectures. Hum Genet 133 (10): 1235–1253. 10.1007/s00439-014-1461-1 24934831

[25] ENCODE Project Consortium (2012) An integrated encyclopedia of DNA elements in the human genome. Nature, 489(7414): 57–74. 10.1038/nature11247 22955616 PMC3439153

[26] Guenther CA, Tasic B, Luo L, Bedell MA, Kingsley DM (2014) A molecular basis for classic blond hair color in Europeans. Nat Genet 46(7): 748–752. 10.1038/ng.2991 24880339 PMC4704868

[27] Koufariotis L, Chen YP, Bolormaa S, Hayes BJ (2014) Regulatory and coding genome regions are enriched for trait associated variants in dairy and beef cattle. BMC Genomics 15: 436 10.1186/1471-2164-15-436 24903263 PMC4070550

[28] Liu DJ, Leal SM (2012) Estimating genetic effects and quantifying missing heritability explained by identified rare-variant associations. Am J Hum Genet. 91(4): 585–596. 10.1016/j.ajhg.2012.08.008 23022102 PMC3484659

[29] An SS, Palmer ND, Hanley AJ, Ziegler JT, Brown WM, Haffner SM, et al (2013) Estimating the contributions of rare and common genetic variations and clinical measures to a model trait: adiponectin. Genet Epidemiol 37(1):13–24. 10.1002/gepi.21685 23032297 PMC3736586

[30] Meuwissen THE, Goddard ME (2010) Accurate prediction of genetic values for complex traits by whole-genome resequencing. Genet 185: 623–631. 10.1534/genetics.110.116590 PMC2881142 20308278

[31] Clark SA, Hickey JM, van der Werf JHJ (2011) Different models of genetic variation and their effect on genomic evaluation Genet. Selec. Evol. 43: 18. 10.1186/1297-9686-43-18 PMC3114710 21575265

[32] Solberg TR, Sonesson AK, Woolliams JA, Odegard J, Meuwissen THE (2009) Persistence of accuracy of genome-wide breeding values over generations when including a polygenic effect. Genet Sel Evol 41: 53 10.1186/1297-9686-41-53 20040081 PMC2813225

[33] Wolc A, Arango J, Settar P, Fulton JE, O’Sullivan NP, Preisinger R, et al (2011) Persistence of accuracy of genomic estimated breeding values over generations in layer chickens. Genet Sel Evol 43: 23 10.1186/1297-9686-43-23 21693035 PMC3144444

[34] Erbe M, Hayes BJ, Matukumalli LK, Goswami S, Bowman PJ, Reich CM, et al (2012) Improving accuracy of genomic predictions within and between dairy cattle breeds with imputed high-density single nucleotide polymorphism panels. J Dairy Sci 95: 4114–4129. 10.3168/jds.2011-5019 22720968

[35] Ober U, Ayroles JF, Stone EA, Richards S, Zhu D, Gibbs RA, et al (2012) Using Whole-genome sequence data to predict quantitative trait phenotypes in *Drosophila melanogaster* . PLoS Genet 8(5): e1002685 10.1371/journal.pgen.1002685 22570636 PMC3342952

[36] MacLeod IM, Hayes BJ, Goddard ME (2014) The effects of demography and long term selection on the accuracy of genomic prediction with sequence data. Genetics 198 (4): 1671–1684. 10.1534/genetics.114.168344 25233989 PMC4256779

[37] Daetwyler HD, Pong-Wong R, Villanueva B, Woolliams JA (2010) The impact of genetic architecture on genome-wide evaluation methods. Genet 185: 1021–1031. 10.1534/genetics.110.116855 PMC2907189 20407128

[38] International HapMap Consortium (2003) The International HapMap Project. Nature 426: 789–796. 14685227 10.1038/nature02168

[39] Saunders EJ, Dadaev T, Leongamornlert DA, Jugurnauth-Little S, Tymrakiewicz M, Wiklund F, et al (2014) Fine-Mapping the HOXB Region Detects Common Variants Tagging a Rare Coding Allele: Evidence for Synthetic Association in Prostate Cancer. PLoS Genet 10(2): e1004129 10.1371/journal.pgen.1004129 24550738 PMC3923678

[40] Van Binsbergen R, Bink MC, Calus MP, van Eeuwijk FA, Hayes BJ, Hulsegge I, et al (2014) Accuracy of imputation to whole-genome sequence data in Holstein Friesian cattle. Genet Sel Evol 46(1): 41. 25022768 10.1186/1297-9686-46-41 PMC4226983

[41] Druet T, Macleod IM, Hayes BJ (2013) Toward genomic prediction from whole-genome sequence data: impact of sequencing design on genotype imputation and accuracy of predictions. Heredity 112(1): 39–47. 10.1038/hdy.2013.13 23549338 PMC3860159

[42] Li H, Durbin R (2009) Fast and accurate short read alignment with Burrows–Wheeler transform. Bioinformatics 25: 1754–1760. 10.1093/bioinformatics/btp324 19451168 PMC2705234

[43] Li H, Handsaker B, Wysoker K, Fennell T, Ruan J, Homer N, et al (2009) The Sequence Alignment/Map format and SAMtools. Bioinformatics 25: 2078–2079. 10.1093/bioinformatics/btp352 19505943 PMC2723002

[44] McKenna A, Hanna M, Banks E, Sivachenko A, Cibulskis K, Kernytsky A, et al (2010). The Genome Analysis Toolkit: a MapReduce framework for analyzing next-generation DNA sequencing data. Genome Res.20: 1297–303. 10.1101/gr.107524.110 20644199 PMC2928508

[45] Grant JR, Arantes AS, Liao X, Stothard P (2011) In-depth annotation of SNPs arising from resequencing projects using NGS-SNP. Bioinformatics 27: 2300–2301. 10.1093/bioinformatics/btr372 21697123 PMC3150039

[46] Purcell S, Neale B, Todd-Brown K, Thomas L, Ferreira MAR, Bender D, et al (2007) PLINK: a toolset for whole-genome association and population-based linkage analysis. Am J Hum Genet 81(3): 559–575. 17701901 10.1086/519795 PMC1950838

[47] Cleveland WS (1979) Robust locally weighted regression and smoothing scatterplots. J Amer Statist Assoc 74: 829–836.

[48] R Development Core Team (2011) R: A language and environment for statistical computing. R Foundation for Statistical Computing, Vienna, Austria ISBN 3-900051-07-0, Available: http://www.R-project.org/.

[49] VanRaden PM, Sullivan PG (2010) International genomic evaluation methods for dairy cattle. Genet Sel Evol 42: 7 10.1186/1297-9686-42-7 20193071 PMC2842236

[50] VanRaden PM, Wiggans GR (1991) Derivation, calculation, and use of national animal model information. J Dairy Sci 74: 2737–2746. 1918547 10.3168/jds.S0022-0302(91)78453-1

[51] Gilmour AR, Gogel BJ, Cullis BR, Thompson R (2009) ASReml User Guide Release 3.0. VSN International Ltd, Hemel Hempstead, HP1 1ES, UK, Available: www.vsni.co.uk.

