# Supplementary figures and images for: Rare Variants in Transcript and Potential Regulatory Regions Explain a Small Percentage of the Missing Heritability of Complex Traits in Cattle
Source: PLoS One. 2015 Dec 7;10(12):e0143945. doi: 10.1371/journal.pone.0143945 (PMC4671594; doi:10.1371/journal.pone.0143945)

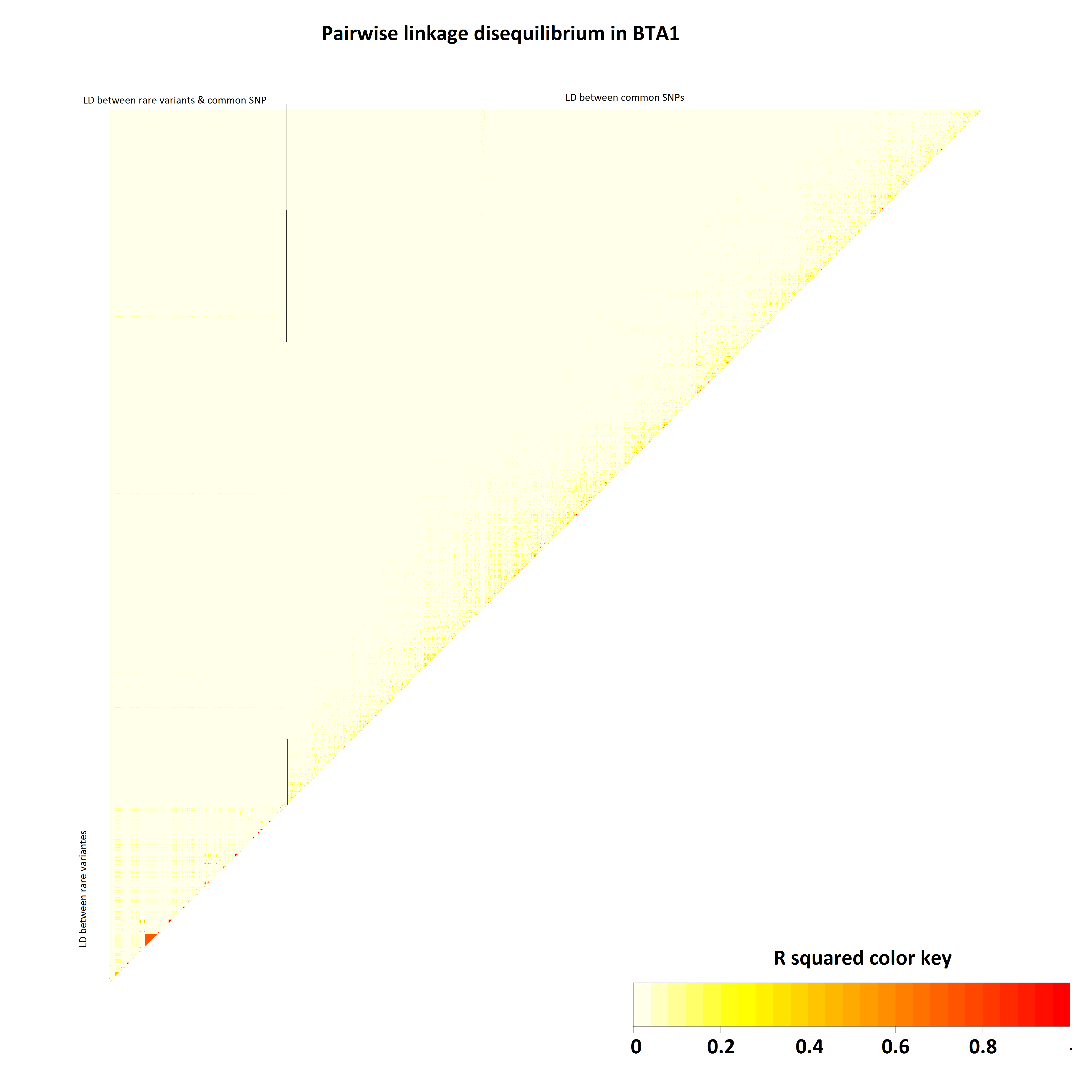

Supplement: S1 Fig — (TIF) [file pone.0143945.s001.tif]

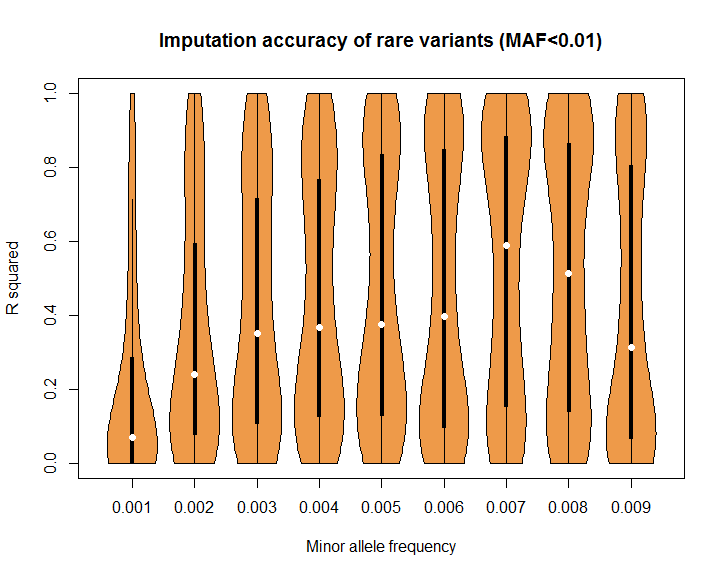

Supplement: S2 Fig — Density distribution for imputation accuracy is shown in the plot for each group of MAF. (TIFF) [file pone.0143945.s002.tiff]

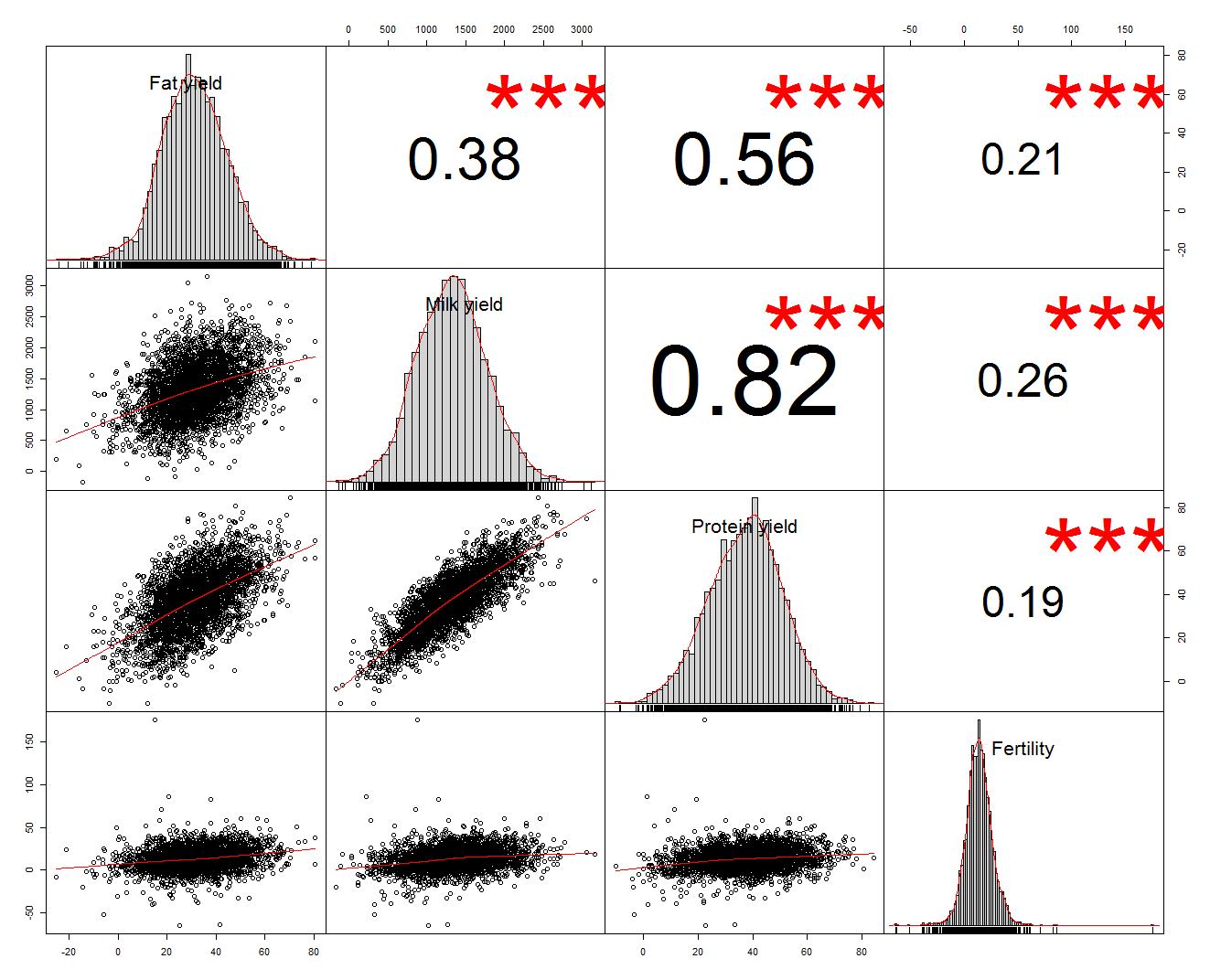

Supplement: S3 Fig — The histograms and corresponding density functions are plotted in the diagonal. On the upper diagonal the value of the phenotypic correlation plus the result of a correlation test (***P<0.001). On the lower diagonal, the bivariate scatterplots, with a fitted line. (TIFF) [file pone.0143945.s003.tiff]
