# Supplementary material for: Rare Variants in Transcript and Potential Regulatory Regions Explain a Small Percentage of the Missing Heritability of Complex Traits in Cattle
Source: PLoS One. 2015 Dec 7;10(12):e0143945. doi: 10.1371/journal.pone.0143945 (PMC4671594; doi:10.1371/journal.pone.0143945)
Supplement: S1 Text — (DOCX) [file pone.0143945.s004.docx]

Rare Variants in Transcript and Potential Regulatory Regions Explain a Small Percentage of the Missing Heritability of Complex Traits in Cattle

**Oscar González-Recio**^1,2^**, Hans D. Daetwyler**^1,2,3^**, Iona M. MacLeod**^1,4^**, Jennie E. Pryce**^1,2,3^**, Phil J. Bowman**^1,2^**, Ben J. Hayes**^1,2,3^**, Michael E. Goddard**^1,4^

# S1 Text. *Probability to validate rare variants with N sire-son duos*.

The probability of validating rare variants given that we observe the WGS data of a sire-son duo was approximated using an exponential function as described next.

For a given variant with alleles *A* and *a* with minor allele frequency equals for the rare allele *P*(a)=*p*, the probability that a sire has at least one rare allele is *P*(*a*_sire_)=*2p*, under Hardy-Weinberg equilibrium. The probability of observing the rare variant in the son given that the rare allele is present in the sire was assumed to be approximately *P*(*a*_son_ | *a*_sire_)=$\frac{1}{2}$. Therefore the probability to observe the rare variant in both the sire and the son is

*P*(*a*_son_ , *a*_sire_)= $\frac{1}{2}$*2p* = *p*.

The probability of validating the rare variant with at least one duo is

$$\begin{matrix} P(\text{at least one} \text{duo} \text{validating} \text{the} \text{rare} \text{variant})= \\ =1-P(\text{no} \text{duo} \text{validating} \text{the} \text{rare} \text{variant})=1-(1-p)^{N} \end{matrix}$$

which can be approximated by $(1-e^{-Np})$.

Assuming that the number of true rare variants with MAF=*p* is *T*, the number of rare variants that we can validate with sire son duos is $T(1-e^{-Np})$.

Hence, we can approximate this function with non-linear regression procedures as

$y=T(1-e^{-Nk})$,

where *y* is the data of validated rare variants with *N* number of duos that were observed in this study, and *T* and *k* are estimated from the data using the R package “nls2” (Grothendieck, 2013), with *k* substituting the allele frequency *p* because many different allele frequencies were observed.

**References**

Grothendieck, G. 2013. nls2: Non-linear regression with brute force. R package version 0.2. http://CRAN.R-project.org/package=nls2
