# Supplementary material for: Rare Variants in Transcript and Potential Regulatory Regions Explain a Small Percentage of the Missing Heritability of Complex Traits in Cattle
Source: PLoS One. 2015 Dec 7;10(12):e0143945. doi: 10.1371/journal.pone.0143945 (PMC4671594; doi:10.1371/journal.pone.0143945)
Supplement: S2 Text — (DOCX) [file pone.0143945.s005.docx]

Rare Variants in Transcript and Potential Regulatory Regions Explain a Small Percentage of the Missing Heritability of Complex Traits in Cattle

**Oscar González-Recio**^1,2^**, Hans D. Daetwyler**^1,2,3^**, Iona M. MacLeod**^1,4^**, Jennie E. Pryce**^1,2,3^**, Phil J. Bowman**^1,2^**, Ben J. Hayes**^1,2,3^**, Michael E. Goddard**^1,4^

**S2 Text. *Pedigree heritability (diagonal), phenotypic covariances (up-diagonal) and pedigree genetic correlations (low-diagonal) for traits in the analyses.***

|  | Fat yield | Milk yield | Protein yield | Fertility |
| --- | --- | --- | --- | --- |
| Fat yield | 0.34 | 2270.9 | 101.3 | 35.9 |
| Milk yield | 0.34 | 0.31 | 5070.0 | 1470.2 |
| Protein yield | 0.53 | 0.82 | 0.33 | 33.6 |
| Fertility | 0.20 | 0.23 | 0.17 | 0.05 |
